# L‐asparaginase doses number as a prognostic factor in childhood acute lymphoblastic leukemia: A survival analysis study

**DOI:** 10.1002/cnr2.1533

**Published:** 2021-08-24

**Authors:** Amanda Cabral dos Santos, Julia Maria Bispo dos Santos, Elisangela da Costa Lima, Marcelo Gerardin Poirot Land

**Affiliations:** ^1^ Instituto de Puericultura e Pediatria Martagão Gesteira ‐ Federal University of Rio de Janeiro, PPGCM – FM (Graduate Program in Medical Clinic –Medical School) Rio de Janeiro Brazil; ^2^ PPGCM – FM (Graduate Program in Medical Clinic –Medical School) Rio de Janeiro Brazil; ^3^ Carlos Chagas Filho Biophysics Institute Federal University of Rio de Janeiro Rio de Janeiro Brazil; ^4^ School of Pharmacy Federal University of Rio de Janeiro Rio de Janeiro Brazil

**Keywords:** asparaginase, doses number, leukemia, survival analysis

## Abstract

**Background:**

The survival of children with acute lymphoblastic leukemia (ALL) has improved due to changes in the treatment and the disease diagnosis. A significant advance was the incorporation of asparaginase. However, hypersensitivity reactions are a common cause of early discontinuation of this drug.

**Aim:**

The proposed study aims to evaluate early interruptions and the influence of the number of asparaginase doses effectively administered on the prognosis of patients with ALL.

**Methods and Results:**

An observational cohort study was carried out, with retrospective data collection, in medical records. The prognostic variables indicated in the protocol applied were used, and the principal outcomes were 5 years event‐free survival (EFS) and 5 years of overall survival (OS) probability. Statistical analyzes were performed using SPPS 20.0 and R. In Cox's proportional hazards model for EFS and OS, variables of prognostic importance (*n* = 126 children) were: high‐risk group (HGR), by the protocol classification, and less than 10 doses of asparaginase. The increased risk of events and death in HGR, who did less than 10 doses, was 3.6 and 7 times, respectively. The study did not show statistical significance for the number of asparaginase doses in patients who were not at high risk.

**Conclusions:**

We demonstrated that the early interruption of asparaginase treatment could negatively impact the prognosis of patients with ALL, especially HGR, reinforcing the need for careful diagnosis of reactions and the availability of alternative types of asparaginase.

## 1 INTRODUCTION

Acute lymphoblastic leukemia (ALL) is the most common type of cancer in childhood, and in Brazil, cancer is the leading cause of death from illness. The ALL survival rates have improved considerably worldwide, having been only 10% in the 1960s and currently reaching 90% survival in 5 years.^1^, ^2^, ^3^

Among the various therapeutic and diagnostic changes over the past decades that have promoted better results for the treatment of ALL, the incorporation of asparaginase into treatment protocols in the 1970s is considered fundamental for the best survival results.^2^, ^4^, ^5^

Asparaginase is an enzyme that hydrolyzes asparagine, an essential amino acid that the leukemic blast cannot synthesize in aspartic acid and ammonia. The first type of asparaginase to be widely used in the treatment of ALL was *Escherichia coli* native asparaginase. However, the high incidence of hypersensitivity reactions (average 30%), which end up causing early treatment interruption, caused others types of asparaginase to replace native asparaginase in many countries.^4^, ^6^

*E. coli*'s PEG‐asparaginase has a chemical conjugation with polyethylene glycol that reduces its immunogenicity and increases the half‐life, thus reducing the number of doses to be administered, making it the first line of treatment in most developed countries. However, although it is less immunogenic than native asparaginase, 5% to 18% of people can develop antibodies against PEG‐asparaginase. For this reason, from 2011, in Europe and the United States, the use of asparaginase from *Erwinia chrysanthemi* was approved, to guarantee the continuity of treatment of people who presented antibodies against PEG‐asparaginase.^6^, ^7^, ^8^, ^9^, ^10^

In Brazil, PEG‐asparaginase was only registered in 2018. The native asparaginase of *E. chrisantemi* remains unregistered with the National Health Surveillance Agency (ANVISA). The unavailability of alternatives for continuing treatment with asparaginase makes many people stop their treatments early. Even in countries where *E. chrisantemi* asparaginase is used as a second line of treatment, this problem can occur due to its shortage or toxicity.^10^, ^11^

As asparaginase is a fundamental medication in the treatment of ALL, the need to use a lower number of doses than recommended in the protocols can directly impact patient survival. Gupta et al,^10^ showed a risk ratio of 1.5 in the analysis of event‐free survival (EFS) for the high‐risk patients compared to those who had their treatment stopped early with those who took all doses.

The proposed study aims at the impact of early interruptions and the influence of the number of asparaginase doses effectively administered on the prognosis of patients with ALL treated at a pediatric teaching hospital located in Rio de Janeiro (Brazil) over 10 years.

## 2 METHODOLOGY

### 2.1 Study design, population and setting

An observational retrospective survival analysis study was carried out in a cohort of Brazilian children with ALL treated between 2005 and 2014.

It included all patients with primary ALL from 2005–2014 (126 children), until 12 years of age at diagnosis, treated at the study hospital. The children have precursor‐B or T immunophenotype. All children have been treated with ALL‐BFM‐IC (International Co‐operative Treatment Protocol for Children and Adolescents with ALL) based protocols with high‐dose Methotrexate (2 g/m^2^ for non‐T‐ALL and 5 g/m^2^ for T‐ALL) and native Asparaginase intravenously. Nursling leukemia patients (<1 year) treated with a specific protocol (Interfant 99 and Interfant 06) were excluded. Two infants, close to 1 year of age, were treated with the ALL‐BFM‐IC and were included in the study. During the study period, children older than 12 years were not treated at the institution.

The IPPMG‐UFRJ (Instituto de Puericultura e Pediatria Martagão Gesteira da Universidade Federal do Rio Janeiro) is a quaternary pediatric teaching hospital with had an oncohematology service for more than 50 years, being one of the four reference sites in the state of Rio de Janeiro for treatment of childhood ALL. The study patients were identified through the high complexity procedure authorization lists (APAC) available at the institution.

### 2.2 Data source and potential predictors

Data were collected in medical records by using a data collection form.

Patients were followed up from diagnosis to the development of an event or until the latest possible date of follow‐up at September 30, 2016. The median follow‐up time of the cohort was 7.2 years.

The prognostic variables of the disease used were those already indicated in the treatment protocol as relevant to the prognosis and available to all patients: (i) sex, (ii) age at diagnosis, (iii) ALL‐BFM‐IC risk factor, (iv) ALL immunological subtype (BCP‐ALL and T‐ALL), (v) WBC at diagnosis, (vi) CNS infiltration status at diagnosis. We collected too the number of asparaginase doses effectively administered during the treatment to each patient.

The main outcomes were 5 years EFS and 5 years of Overall Survival probability.^12^

### 2.3 Statistical analyses

A Kaplan–Meier (KM) survival analysis of the study population was performed based on the data collected. The primary outcomes were EFS, defined as the time (in years) from diagnosis to the first event (relapse, death, or secondary neoplasia), and overall survival (OS), defined as the time (in years) from diagnosis to death.

Patients not experiencing an event were censored at the time of the last contact with the cancer center or at the latest possible date of follow‐up on September 30, 2016.

Univariate and multivariable analysis were conducted using time‐to‐event techniques (Cox proportional hazard models),^13^ which allowed the identification of the hazard ratio (HR) for the analysed variables. The Martingale residuals analysis determined the cutoff point for the number of asparaginase doses. The PH assumption was verified through the Schoenfeld residuals test. Variables were included in the multivariable analysis if associated with *p* values <.2 in univariate analysis, and no significant collinearity was observed between the variables to be included. We used the backward elimination (using the likelihood ratio test) to obtain a parsimonious predictive model. Analyzes were performed using SPPS 20.0 and R.

## 3 RESULTS

### 3.1 Study cohorts

Between 2005 and 2014, 126 ALL children treated with modified ALL‐BFM‐IC 2002 or 2009 were identified. The distribution of demographic and prognostic variables identified in the disease diagnosis is shown in Table 1.

**TABLE 1:** Demographic and prognostic variables at diagnosis (Rio de Janeiro, 2005–2014)

Variable	(*N* = 126)	(%)
Sex		
Female	55	43.7
Male	71	56.3
Age (years)		
<1	2	1.6
1–5	75	60.0
6–9	36	28.8
≥10	12	9.6
Leucocyte count		
<10 000	50	39.7
10 000–49 999	41	32.5
50 000–99 999	10	7.9
≥100 000	25	19.8
Immunophenotype		
Non‐T	107	84.9
T	19	15.1
CNS +	5	3.9

Abbreviations: CNS, central nervous system; Non‐T, B precursors ALL; T, T‐ALL.

Table 2 summarizes the main treatment outcomes and occurrences of the following children.

**TABLE 2:** Treatment outcomes and occurrence (Rio de Janeiro, 2005–2014)

Outcomes	(*N* = 126)
Induction phase	
Death	3 (2.4%)
Treatment abandonment	0
After induction phase	
Resistant disease	6 (4.8%)
D33 complete remission	120 (95.2%)
Treatment abandonment	0
CR death	9 (7.14%)
Relapses	31 (24.6%)
CCR	84 (66.7%)

Abbreviations: CCR, continued complete remission; CR, complete remission; D33, 33th day after beginning of treatment.

### 3.2 Predictors of event free survival

KM EFS analysis results (Table 3) demonstrated the candidate variables (*p* < .20) for multivariate analysis were ALL‐BFM‐IC risk group, number of administered asparaginase doses, sex, leucocyte count and D8 prednisone response.

**TABLE 3:** KM analysis of risk factors for event‐free survival (EFS)

Variables	*N*	%	6 years de EFS	plogrank
ALL	126	100	68.8	
Sex				
Male	71	56.3	62.4	0.099
Female	55	43.7	76.0	
Age				
<1 or ≥10 years	11	8.7	75.5	0.638
≥1 and <10 years	115	91.3	67.6	
Leucocyte count				
<50 000/mm^3^	91	72.2	72.4	0.039
≥50 000/mm^3^	35	27.8	57.2	
Immunophenotyping				
Non‐T	107	84.9	66.3	0.389
T	19	15.1	78.3	
D8 Prednisone response				
<1000/mm^3^	93	73.8	73.1	0.043
≥1000/mm^3^	33	26.2	54.8	
L‐asparaginase doses				
<10 doses	80	63.5	58.9	0.003
≥10 doses	46	36.5	83.9	
BFM risk group				
SRG	27	23	88.1	0.005
IRG	43	36	74.0	
HRG	49	41	55.3	

Abbreviations: D8, 8th day after beginning of treatment; HRG, high risk group; IRG, intermediate risk group; Non‐T, B precursors ALL; SRG, standard risk group; T, T‐ALL.

Table 4 presents the parsimonious predictive model obtained through the Cox proportional hazards multivariate analysis for the factors identified as candidate variables.

**TABLE 4:** Predictive model for EFS

Variable	HR	CI (95%)	*p*
BFM risk group			
SRG	1		.002
IRG	1.707	0.467–6.234	.418
HRG	5.065	1.505–17.042	.009
<10 L‐asparaginase doses	3.548	1.519–8.285	.003

Abbreviations: CI, confidence interval; HR, hazard ratio; HRG, high risk group; IRG, intermediate risk group; SRG, standard risk group.

In the HRG patients, the parsimonious predictive model obtained through the Cox proportional hazards multivariate analysis, the only identified prognostic factors was the use of less than 10 L‐asparaginase doses (HR = 3.603 ‐ CI: 1.317–9.861, *p* = .013).

### 3.3 Predictors of overall survival

The KM OS analysis, shown in Table 5, demonstrated that candidate variables (*p* < .20) for multivariate analysis were BFM Risk Group, number of administered asparaginase doses and leucocyte count.

**TABLE 5:** KM analysis of risk factors for overall survival (OS)

Variables	*N*	%	6 years OS	plogrank
ALL	126	100	71.8	
Sex				
Male	71	56.3	69.4	0.443
Female	55	43.7	75.3	
Age				
<1 or ≥10 years	15	8.7	77.4	0.772
≥1 and <10 years	111	91.3	71.1	
Leucocyte count				
<50 000/mm^3^	91	72.2	73.2	0.039
≥50 000/mm^3^	35	27.8	60.0	
Immunophenotyping				
Non‐T	107	84.9	70.5	0.616
T	19	15.1	78.0	
D8 prednisone response				
<1000	93	73.8	74.1	0.338
≥1000	33	26.2	64.9	
L‐asparaginase doses				
<10 doses	46	36.5	58.1	<0.001
≥10 doses	80	63.5	90.6	
BFM risk factor				
SRG	27	22.7	96.2	0.001
IRG	43	36.1	74.4	
HRG	49	41.2	57.9	

Abbreviations: D8, 8th day after beginning of treatment; HRG, high risk group; IRG, intermediate risk group; Non‐T, B precursors ALL; SRG, standard risk group; T, T‐ALL.

Table 6 presents the parsimonious predictive model obtained through the Cox proportional hazards multivariate analysis for the factors identified as candidate variables.

**TABLE 6:** Predictive model for OS

Variable	HR	CI	*p*
BFM risk group			
SRG	1		.001
IRG	4.569	0.578–36.138	.150
HRG	15.716	2.096–117.847	.007
<10 L‐asparaginase doses	6.334	2.163–18.547	.001

Abbreviations: HRG, high risk group; IRG, intermediate risk group; SRG, standard risk group.

In the HRG patients, according to the parsimonious predictive model obtained through the Cox proportional hazards multivariate analysis, the only identified prognostic factor was the use of less than 10 L‐asparaginase doses show a 6.978 hazard ratio (CI: 2.031–23.970, *p* = .002).

### 3.4 L‐asparaginase doses number

The total number of L‐asparaginase doses effectively received during the treatment by the HRG patients and the other risk groups were not significantly different (median 9 (IQR = 4) vs. 8 (IQR = 4) doses, *p* = .207). Nonetheless, the L‐asparaginase dose number was the most important prognostic factor in HRG patients. This study did not indicate a prognostic significance of L‐asparaginase dose number for the non‐HRG patients.

## 4 DISCUSSION

In the study population, there was a slight predominance of boys (56.3%), as well as more children from 1 to 5 years (60%). This slight difference in the incidence of ALL in boys (57.1%), as well as the greater number of cases in the age group from 1 to 5 years old (78%) was also observed by Stary et al,^12^ in the ALL BFM‐IC 2002 clinical trial.

The leucocyte count, the immunophenotype, the early response to prednisone are also important prognostic factors, used for the classification of low, moderate, or high risk of the disease. In the study population, we observed that these characteristics had a distribution similar to that of other studies, with a predominance of leucocyte counts below 50 000/mm^3^ (72.2%), non‐T immunophenotype (84.9%), and early response to prednisone in most cases (73.8%). However, in the present study, a high percentage (41.2) of high‐risk cases was observed, differing from that observed by Stary et al,^12^ 17%.

Death during the induction of remission and in complete remission reached 9.5% of the treated children in our study, and 7.2% of the children in the clinical trial of ALL IC‐BFM 2002. There was no abandonment of treatment neither during nor after induction of remission, which was observed after work to raise awareness of the importance of complete treatment with families (10–15 years ago, in the study setting). The cases of resistant disease and relapses were higher than those found in the ALL BFM 2002‐IC (4.8 vs. 0.6%) and (24.6 vs. 16.4%), respectively. Such differences may be due to the greater number of high‐risk cases observed in the present study, as well as the size of the observed population, since the ALL IC‐BFM 2002 is a multicenter study with 5060 children followed up in several countries.^12^

When comparing EFS in the present and study and in the work of Stary et al,^12^ we can see they are very close according to the risk classification. For SRG children, we found 88.1% of 5‐years EFS versus 90%–81% 5‐years EFS from Stary et al; IRG, 74% versus 83%–75%; and HRG, 55.3% versus 62%–55%, respectively. Stary et al^12^ had two treatment arms, so two different survivals.

The multivariate analysis of EFS time showed that in addition to ALL‐BFM‐IC risk groups, the number of asparaginase doses (<10 doses) was also a variable to be considered in a parsimonious predictive model of risk factors. However, only high‐risk patients had a higher risk of events and death with early discontinuation of asparaginase treatment due to an adverse reaction. As in the present study, Gupta et al^10^ observed an increased risk of events in high‐risk patients who were unable to complete their treatment with asparaginase. According to these authors, the discontinuation of asparaginase use leads to an increase of 50% in the hazard of an event on high‐risk patients and a non‐increased risk for non‐high‐risk patients. They do not mention the number of doses from which the increased risk is observed.

Pui^14^ also did not identify an association between the number of doses and the increased risk of events in non‐high‐risk patients. They concluded that Erwinia's asparaginase might not be necessary for patients who made at least 50% of the scheduled doses. This work indicates, like Pui^14^ work, that Erwinia's asparaginase may not be necessary for non‐HRG patients that have not completed the scheduled doses. The average of doses administered was eight for this group of patients, compatible with the number of induction doses of remission on ALL‐BFM‐IC protocol. This stage completion seems fundamental, although the overall and EFS does not seem to be affected by reinduction doses omission for non‐high‐risk patients, in case of adverse reaction, for example.

Such a difference in the influence of the number of doses, from where higher risks are observed for a group of patients (high risk) and not for others (not high risk) corroborates what was indicated by previous studies. A subgroup of patients with a better prognosis of the disease seems to obtain a favorable result with minimal use of asparaginase, as long as they receive other effective systemic therapies.^10^ No standard‐risk patient developed allergic hypersensitivity during induction of remission high doses or extensive asparaginase regimens may not be necessary for the standard‐risk patient.^14^

*E. chrisantemi* asparaginase, when available, is used as an alternative for continuity of treatment when there is an allergic hypersensitivity reaction or silent inactivation of *E. coli*‐derived asparaginase. Patients who received Erwinia's asparaginase maintained EFS. This demonstrated the urgency of Erwinia's asparaginase's global availability or the development of alternative recombinant forms of asparaginase.^10^ We confirmed in the present study that for high‐risk patients, especially those who are unable to take at least 10 doses, continuity of treatment with Erwinia's asparaginase would be essential.

In Brazil, the country where the present study was carried out, the alternative to asparaginase derived from *E. coli* (native and PEG‐asparaginase) is not available. Therefore, once a hypersensitivity reaction is observed, treatment with asparaginase is definitely suspended.

Reactions related to asparaginase infusion are the leading cause of early treatment interruption since allergic hypersensitivity reactions are associated with the formation of neutralizing antibodies that make it impossible to continue treatment with the type of asparaginase used, being indicative of resistance to treatment. Pancreatitis may cause the early termination of asparaginase treatment without a possible change of type of asparaginase formulation. Thrombosis can lead to delays in treatment, as it requires temporary interruption of therapy, according to ALL‐BFM‐IC protocol.^6^, ^12^, ^15^, ^16^

However, not all infusion reactions to asparaginase require the interruption of treatment with asparaginase. Non‐allergic hypersensitivity reaction and the hyperammonemia reaction can also occur during the asparaginase infusion. Recent works highlighted careful monitoring and correct classification of reactions for kind and grade so that a treatment interruption or exchange for an alternative type of asparaginase is performed unnecessarily.^17^, ^18^, ^19^, ^20^ It is imperative to avoid unnecessary early interruptions, and to guarantee alternatives to asparaginases derived from *E. coli* when necessary.

We corroborate the different impacts of early discontinuation of asparaginase treatment according to the ALL risk classification and determine the minimum number of doses (10). High‐risk children start to have a worse prognosis when this drug has to be interrupted without the minimum of 10 doses. However, for non‐high‐risk patients, it was not possible to determine this number of doses accurately. We infer that if, on average, all patients in the cohort took at least all the induction doses, complete induction is the minimum necessary for this subgroup of patients. This study followed patients using *E. coli* native asparaginase, the only type of asparaginase available in Brazil at the time of this investigation. Studies with a pegylated form are needed.

## 5 CONCLUSION

High‐risk and non‐high‐risk patients have been shown to have a different impact on global and EFS for the early discontinuation of asparaginase treatment. The first group needs to use at least 10 doses of *E. coli* native asparaginase. On the other hand, for the second, induction completeness is necessary, but when the reaction is after this phase of treatment, no decrease in survival was identified due to early interruption.

These findings reinforce the need for differential diagnosis of adverse reactions that do not prevent the asparaginase use and the availability of other formulations of this enzyme for high‐risk LLA treatment, especially in developing countries.

## CONFLICT OF INTEREST

We declare the absence of conflicts of interest of a personal, commercial, academic, political, or financial nature and that the manuscript has not been submitted elsewhere nor previously published.

## AUTHOR CONTRIBUTIONS

**Amanda Cabral dos Santos:** Conceptualization (supporting); formal analysis (lead); investigation (lead); methodology (supporting); writing – original draft (lead). **Julia Maria Bispo dos Santos:** Formal analysis (supporting); investigation (supporting); writing – review and editing (lead). **Elisangela da Costa Lima:** Conceptualization (lead); data curation (lead); formal analysis (supporting); methodology (supporting); project administration (supporting); writing – review and editing (lead). **Marcelo Gerardin Poirot Land:** Conceptualization (lead); formal analysis (lead); investigation (lead); methodology (lead); supervision (lead); writing – review and editing (supporting).

## ETHICS STATEMENT

The research project was submitted to and approved by the Research Ethics Committee of the Pediatrics Institute of the Federal University of Rio de Janeiro (2.787.465) Retrospective secondary data was collected without any interaction between researchers and children or parents. Personal information of the participants was kept blinded to investigators. Waiver of parental permission (Written informed consent from the participant's legal guardian) was requested and authorized by REC.

## Data Availability

The data that support the findings of this study are available from the corresponding author upon reasonable request.
